# Clinical Report from Medical Wards of Mercy Hospital

**Published:** 1868-10

**Authors:** N. S. Davis

**Affiliations:** Professor of Practical and Clinical Medicine, in Chicago Medical College


### The Clinique.

### CLINICAL REPORT FROM MEDICAL WARDS OF MERCY HOSPITAL.

By N. S. DAVIS, M.D., Professor of Practical and Clinical Medicine, in Chicago Medical College.

### TUBERCULOSIS AND CHRONIC DIARRHŒA.

The Professor remarked, that the first case to which he should invite the attention of the Class was a young man, aged 18 years; naturally slender, light complexion; belonging to the laboring class, who was admitted to the Hospital only two or three days previously, but who had been sick about six months. The patient states that most of the time since the commencement of his sickness he has had tenderness, swelling, and pain in the left half of the abdomen; and at times fever and cough. He thinks that there existed a tumor in the left side of the abdomen, which at a certain stage of his disease broke, and discharged into the bowels, giving rise to purulent discharges by rectum. Since which time, he has continued to have muco-purulent discharges, sometimes mixed with blood, and resembling those of chronic dysentery.

Such are the chief items in the history of the case, as stated by the patient himself. The attention of the Class was then called to the present condition of the patient. The anæmic hue of the skin; the thin and slightly retracted lips; the expression of anxiety; the general emaciation; the quick, irritable pulse; connected with the absence of febrile heat and the length of time that the patient had been sick, clearly indicated pathological changes somewhere of a serious nature. The continuance of muco-purulent discharges from the bowels every three or four hours, and sometimes more frequently, with still existing tenderness over the sigmoid flexure of the colon, point directly to the abdomen as the seat of the more advanced pathological changes.

Is the case one of simple chronic recto-colitis or dysentery, involving thickening and ulceration of the mucous membrane of the descending colon? Or is this state of the mucous membrane complicated with disease of the mesenteric glands? To determine these questions requires a careful physical examination of the chest and abdomen. The latter is moderately tympanitic, with some tenderness towards the left side, but neither palpation nor percussion enables us to detect any well-marked tumors or enlarged glands. This, however, does not prove the non-existence of disease in the mesentery; for when the abdomen is full as in the present case, it is impossible to trace out by the touch even considerable enlargements of the glands in the mesentery.

Attention was next turned to the chest, and after the Class had listened carefully to the pulmonary and cardiac sounds, it was found that the respiratory murmur was harsh and irregular over the upper lobes of both lungs, with marked increase of vocal vibration, and moderately diminished resonance on percussion.

These physical signs plainly indicate increased density of the upper part of the lungs; and the absence of all the usual symptoms of pneumonia render it almost certain that the increased density is caused by crude tubercular deposits.

If this is true, it renders it highly probable that more or less of the tubercular deposits also exist in the follicles of the mucous membrane of the larger intestines, causing the protracted and obstinate intestinal discharges. The general aspect of the patient, the quick pulse, the results of physical examination of the chest, and the long continuance of the intestinal disease, in spite of protracted and judicious treatment (he had been treated several weeks in another hospital before admission here), leave little doubt but the case is one of tuberculosis. This has a very important bearing on the prognosis. Simple chronic dysentery, even of long standing, can generally be cured. But chronic dysentery or diarrhœa founded on tuberculosis is very generally fatal. The condition of the patient may be temporarily improved, by lessening the frequency of the discharges, and regulating judiciously the diet. But the improvement is only temporary. Even if, as now and then happens, the intestinal discharges entirely cease, or become natural, in a few weeks cough supervenes, and all the active symptoms of pulmonary tuberculosis are developed.

The treatment in all these cases must be tonic and anodyne; the one to sustain the general tone of the system, and the other to allay the local irritations. In similar cases, it was stated that a combination of sub. nit. bismuth, sub. carb. ferri, and morphine, had been found of great value.

Accordingly, the following prescription was directed:

℞. Sub. Nit. Bismuth,-------------------- ʒiij.

Sub. Carb. Ferri,----------------------- ʒij.

Sulph. Morph.,------------------------ 5 grs.

Mix, divide into 30 powders; and give one before each meal-time and at bedtime.

If this did not sufficiently restrain the discharges, the doses may be given more frequently. The diet must be very simple, yet nutritious; such as milk, milk and flour, boiled rice, and animal broths.

PULMONARY TUBERCULOSIS, WITH PARTIAL PARALYSIS OF THE ARMS.

The next case to which the attention of the Class was directed was that of a young man, who was admitted into the Hospital about three months previously, in an advanced stage of tubercular phthisis. He was a native of Germany, about 25 years of age, and had been declining in health for six or eight months.

At the time of admission he was emaciated; pale; pulse frequent and weak; skin cool, with copious night-sweats; frequent serous discharges from the bowels; cough and purulent expectoration. He was too feeble to leave his bed.

Auscultation and percussion revealed extensive tubercular deposits in both lungs, and a large suppurating cavity in the middle lobe of the left side. The latter still exists, and affords an excellent sample of the cavernous sounds to which the Class had an opportunity of listening with the stethoscope. To check the hectic fever and night-sweats, as well as to lessen the diarrhœa, he was placed, immediately after admission into the Hospital, on the use of the powders of sub. nit. bismuth, sub. carb. ferri, and sulph. morph., every 4 hours, with sweet milk and wheat-flour porridge for nourishment.

For some time he continued very feeble, affording every indication of an early fatal result. But after the first two weeks the condition of the bowels and digestive organs began to improve slowly; and for the last three weeks they have been quite regular, with a fair appetite. The hectic and night-sweats have also ceased, but he still coughs and expectorates muco-purulent matter, more especially every morning. His breathing is very short, and slight exercise induces panting and palpitations. His feet and ankles are quite anasarcous, with slight evidences of œdema over the whole surface of the body.

But the point of special interest connected with this case was a partial paralysis of motion in both upper extremities. He could not elevate the arms to a position on a level with the shoulders, and found great difficulty in feeding himself. It illustrated a class of cases of paralysis arising from deficient oxygenation and decarbonization of the blood, by which it becomes incapable of maintaining the natural action of the nervous centres, and greatly favors the fatty degenerations of tissue. In two or three cases of a similar character, except there had been no tendency to diarrhœa, the internal use of 10-grain doses of chlorate of potassa dissolved in mucilage of gum-Arabic, and repeated four times a day, had done much good. The extreme sensitiveness of the bowels, in the present case, renders the chlorate inadmissible. The remedies most likely to benefit the case before us, in addition to the tonic and anodyne powders before mentioned, are electricity and small doses of strychnia.

The Lecture closed by directing the patient to have gentle currents from an electro-magnetic battery passed through the arms and spine, once every day; and a sugar-coated pill of the thirty-second part of a grain of strychnia after each meal; continuing the powders of bismuth, iron, and morphia before each meal and at bedtime, as before directed.

Note.—Two weeks have elapsed since the foregoing clinic, and the last patient mentioned is up, dressed, and out in the open air a little every day, and the ability to use the arms has improved.

